# Identification of SH3 domain interaction partners of human FasL (CD178) by phage display screening

**DOI:** 10.1186/1471-2172-10-53

**Published:** 2009-10-06

**Authors:** Matthias Voss, Marcus Lettau, Ottmar Janssen

**Affiliations:** 1 Institute of Immunology, Christian-Albrechts-University of Kiel, D-24105 Kiel, Germany; 2 Current address : Adolf-Butenandt-Institute, Ludwig-Maximilians University, D-80336 Munich, Germany

## Abstract

**Background -:**

Fas ligand is a cytotoxic effector molecule of T and NK cells which is characterized by an intracellular N-terminal polyproline region that serves as a docking site for SH3 and WW domain proteins. Several previously described Fas ligand-interacting SH3 domain proteins turned out to be crucial for the regulation of storage, expression and function of the death factor. Recent observations, however, indicate that Fas ligand is also subject to posttranslational modifications including shedding and intramembrane proteolysis. This results in the generation of short intracellular fragments that might either be degraded or translocate to the nucleus to influence transcription. So far, protein-protein interactions that specifically regulate the fate of the intracellular fragments have not been identified.

**Results -:**

In order to further define the SH3 domain interactome of the intracellular region of Fas ligand, we now screened a human SH3 domain phage display library. In addition to known SH3 domains mediating binding to the Fas ligand proline-rich domain, we were able to identify a number of additional SH3 domains that might also associate with FasL. Potential functional implications of the new binding proteins for the death factor's biology are discussed. For Tec kinases and sorting nexins, the observed interactions were verified in cellular systems by pulldown experiments.

**Conclusion -:**

We provide an extended list of putative Fas ligand interaction partners, confirming previously identified interactions, but also introducing several novel SH3 domain proteins that might be important regulators of Fas ligand function.

## Background

Protein-protein interactions link signal transduction pathways from receptors to the nucleus and govern intracellular processes as diverse as organelle targeting, directional transport, cytoskeletal reorganization, membrane positioning, endo- and exocytosis and protein degradation. Protein-protein interactions are mostly mediated by modular domains with the best studied examples being Src homology (SH) 2 and 3 domains [1]. SH3 domains are phylogenetically highly conserved protein interaction modules that comprise 50 to 70 amino acids and are found in a variety of functionally unrelated proteins. As typical interaction modules, they fold into a similar globular structure. Most SH3 domains bind proline residues in a certain arrangement (e.g. PxxP) in so-called "proline-rich domains" (PRD) [2,3].

Fas ligand (FasL, CD95L, Apo-1L, CD178) is a type II transmembrane protein of the tumor necrosis factor superfamily that acts as a prototypic death factor of immune cells [4,5]. FasL is employed by cytotoxic T lymphocytes (CTLs) and natural killer (NK) cells to kill virus-infected or tumorigenic cells. It is implicated in the downregulation of immune responses by activation-induced cell death, the establishment of immune privilege, and in the modulation of T cell activation [6-8]. FasL is stored in so-called secretory lysosomes and is recruited to the immunological synapse in an activation-restricted fashion. Its surface expression is down-modulated by shedding through a disintegrin and metalloprotease (ADAM) 10 activity and intramembrane proteolysis by the γ-secretase-like protease SPPL2a (signal peptide peptidase-like 2a). The released intracellular domain may translocate to the nucleus or be prepared for degradation [9-11].

**Figure 1:**
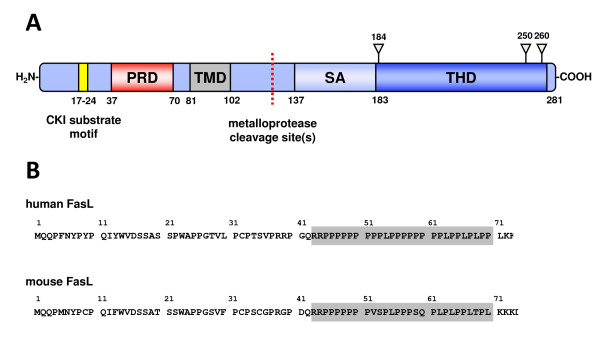
**Schematic representation of FasL and its proline-rich region**. **A**. FasL is a type II membrane protein. In its cytoplasmic N-Terminus, it contains a casein kinase I (CKI) substrate motif and a proline-rich domain (PRD). C-terminal to its transmembrane region (TM), FasL harbors cleavage sites for metalloproteases, a self-assembly (SA) region required for trimerization, several glycosylation sites and the C-terminal receptor binding TNF homology domain (THD). **B**. The amino acid sequences for the N-terminal cytoplasmic region of human and murine FasL are displayed to highlight the unique proline-rich domain spanning about 30 amino acids.

The FasL N-terminus comprises a unique PRD that contains several putative SH3 domain binding sites (Fig 1). Different experimental approaches have already led to the identification of several FasL-interacting proteins including Src-related tyrosine kinases (Fyn, Lyn, Lck, Hck, Fgr, Src, and Abl), adapter proteins involved in T cell receptor (TCR)-associated signal transduction (Grb2, Gads, p85 subunit of PI3 kinase, Nck) and members of the Pombe Cdc15 homology (PCH) protein family (protein kinase C and casein kinase substrate in neurons 1-3 (PACSIN1-3), Formin-binding protein 17 (FBP17), Cdc42-interacting protein 4 (CIP4), CD2-binding protein 1 (CD2BP1), Rho GTPase-activating protein 4 (ARHGAP4), Fer/CIP4-homology (FCH) and double SH3 domains 1 (FCHSD1) and SLIT-ROBO Rho GTPase-activating protein 2) [12-16]. Many aspects of FasL biology are indeed closely linked to PRD-SH3 domain interactions: members of the PCH family regulate lysosomal association [16,17], tyrosine kinases are involved in reverse signaling and sorting of human FasL to multivesicular bodies [18,19], and the adapter protein Nck is crucial to bring FasL loaded vesicles to the immunological synapse [20].

The aim of the present study was to get an idea about the complete FasL-SH3 domain interactome and to define interactors that could be involved in the translocation of FasL to the nucleus or in the priming of N-terminal fragments (NTF) for degradation. We used a phage display library that covers the entire human SH3 domain proteome to screen for interactions with the FasL N-terminus. We confirmed several previously identified interactions and introduce a number of SH3 domain proteins as novel candidate FasL binding partners. These include additional non-receptor tyrosine kinases (e.g. the Tec kinases), sorting nexins and other cytosolic or nuclear adapter proteins that could be involved in the intracellular trafficking of FasL or FasL fragments.

## Methods

### Production of a recombinant GST-hFasL fusion protein

Affinity purification of Glutathione S-transferase (GST) and the GST-hFasL(1-80) fusion protein was performed as previously described [14]. Briefly, an overnight culture of transformed bacteria was diluted in fresh LB-Amp medium and incubated for 1.5 h before induction of protein expression by 1 mM isopropyl-β-D-1-thiogalactopyranoside (IPTG) for 4 h. Bacteria were pelleted, re-suspended in PBS and lysed by sonification and addition of 1% (v/v) Triton X-100. Cell debris was removed by centrifugation and Glutathion 4B sepharose beads (GE Healthcare, Little Chalfont, UK) were added to the clear lysate and rotated at 4°C. For elution of bound fusion protein, reduced glutathion was used. The eluates were concentrated and washed with PBS by centrifugation in Amicon^® ^filter units (Millipore, Billerica, USA).

### Bacteria and Phages

Heat-shock competent *E. coli *DH5α (Invitrogen Carlsbad, USA) were propagated according to standard procedures. The *E. coli *strain TG1 was from Stratagene (La Jolla, USA). Isolated colonies of *E. coli *TG1 were picked from M9 plates and expanded in M9 plus medium with high glucose. The next day, the equivalent of OD (λ = 600 nm) = 0.05 was used to inoculate 50 ml sterile LB medium and bacteria were cultivated at 37°C. Transduction competence of *E. coli *TG1 cells was checked by infection with M13K08 helper phages (New England Biolabs, Ipswich, USA) conferring kanamycin resistance. For the screening of SH3 domains binding to FasL, we used the "All SH3 Domain Phager" library from GeneArt (Regensburg, Germany). In this library, expression-optimized coding sequences from all conventional human SH3 domains are inserted in frame to the coat protein pVIII ORF into the phagemid vector pG8JH by BssHII- and NotI-mediated restriction. As a selection marker, the corresponding phagemid vectors contain a β-lactamase gene.

### Phage display library screening

GST-hFasL(1-80) fusion protein or GST as a control were covalently bound to epoxy-functionalized magnetic beads following the provided protocol (Dynabeads^® ^M-270 epoxy, Invitrogen). For all washing and separation steps, the beads solution was placed in a magnetic bead separator (Dynal/Invitrogen) and unbound supernatants were carefully removed. Binding of the protein to the beads was done overnight at 4°C with slow rotation. The protein solution was removed, blocking buffer was added, and the beads were incubated at room temperature for 1 h. The beads were washed extensively with PBS and finally re-suspended in 200 μl phage solution (6 × 10^9 ^plaque-forming units (PFU)) with blocking buffer and incubated at room temperature. After binding of phages to beads (1.5 h at room temperature), samples were washed extensively. The supernatant was removed and phages were eluted from the beads. This solution was neutralized and phage eluates were serially diluted with sterile LB medium. For each transduction, 500 μl TG1 cells were shaken at 37°C for 10 min. After addition of 5 μl diluted phages, the samples were briefly vortexed and incubated for 30 min at 37°C without shaking. 100 μl of each sample were then plated on SOBAG plates and incubated overnight at 37°C. Colonies were counted and phage titers in eluates were determined. In order to optimize the binding specificity, low (0.15%) and high (0.50%) concentrations of Tween-20 in washing and blocking buffers were compared.

### Preparation of phagemid DNA, sequencing, and identification of interacting SH3 domains

*E. coli *clones were propagated in 5 ml LB-Amp medium. Phagemid DNA was isolated using NucleoSpin^® ^(BD, Franklin Lanes, USA) or QIAPrep^® ^(Qiagen, Hilden, GER) kits following the manufacturer's protocols. SH3 domain coding regions were sequenced using J-55 (5'-CCTATTGCCTACGGCAGCC-3') and H-301 (5'-CAGGGAGTCAAAGGCCGCTTTTGC-3') sequencing primers and the BigDye^® ^Terminator v1.1 cycle sequencing kit (Applied Biosystems/Invitrogen). Samples were analyzed by capillary electrophoresis (Genetic Analyser 3130, Applied Biosystems/Hitachi). Sequence analysis was done using the VectorNTI 10 software suite (Invitrogen). Individual sequences were translated *in silico *and SH3 domains were identified by BLAST using Swiss-Prot servers.

### Verification of selected SH3 domain-FasL interactions

For verification of the Itk SH3 domain-FasL interaction, pulldown experiments using recombinant GST-SH3 domain fusion proteins were performed from stably FasL expressing KFL9 and JFL39.1 cell lines as described previously [20]. Interactions of full length SNX9, SNX18 and SNX33 with FasL were verified in HEK 293 T cells. To this end, cells were transfected with HA-tagged expression constructs of the respective sorting nexins (kindly provided by S. F. Lichtenthaler, German Center for Neurodegenerative Diseases (DNZE) - Munich & Adolf-Butenandt-Institute, LMU Munich) by calcium phosphate precipitation. 18 h post transfection, cells were lysed in NP-40 lysis buffer supplemented with protease inhibitors. Lysates were subjected overnight to precipitation with GST-hFasL(1-80) fusion protein and glutathione sepharose beads. Beads were washed four times with NP-40 lysis buffer and subjected to electrophoresis and Western blot using anti-HA mAb 3F10 (Roche Applied Science, Basel, Switzerland).

## Results and discussion

In its proline-rich domain (PRD), the human FasL contains several putative SH3 domain binding sites. Previous studies have employed different strategies to screen for FasL interaction partners, including yeast two-hybrid assays, peptide- and recombinant SH3 domain-based experiments as well as proteomic screens of FasL precipitates from T cells and neuronal cells [12-16]. We now screened a human SH3 domain phage display library, covering the entire human SH3 proteome with 288 conventional SH3 domains.

In order to obtain phage clones binding the FasL N-terminus, magnetic beads were coated with affinity-purified GST-hFasL(1-80) fusion protein or GST as a control. The beads were used to pan the phage display library as described. Following binding, the beads were extensively washed with buffer containing either 0.15% or 0.5% Tween-20. It is important to mention that phage titers differed significantly depending on the detergent concentration. For less stringent washing conditions (0.15% Tween-20), a titer of (2.85 ± 0.15) × 10^7 ^PFU/ml was calculated in eluate obtained from the GST-panning, while the titer following more stringent washing was (0.185 ± 0.075) × 10^7 ^PFU/ml. Under the same conditions, eluates from GST-hFasL(1-80) panned beads displayed titers of (1.59 ± 4.53) × 10^8 ^PFU/ml and (3.31 ± 0.6) × 10^7 ^PFU/ml, respectively, showing that the specificity of phage-displayed SH3 domain-pVIII fusion proteins binding to the hFasL(1-80) moiety was significantly increased at higher stringency.

**Figure 2:**
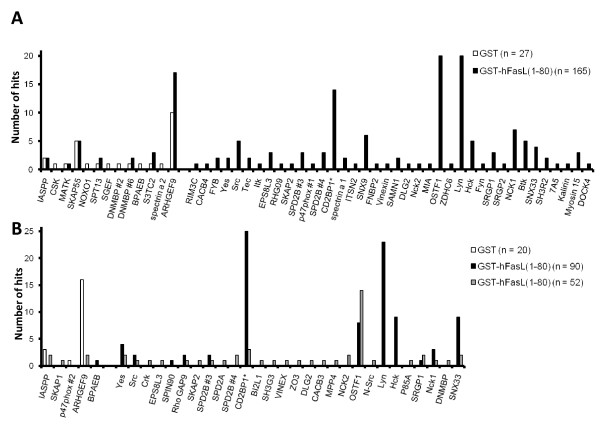
**Identification of FasL-interacting SH3 domains**. Phagemid DNA from transduced clones was isolated and sequenced to unambiguously identify the SH3 domains responsible for interactions with glutathione S-transferase (GST) (white) and GST-hFasL(1-80) (black and grey), respectively. SH3 domains binding unspecifically to the GST moiety were identified by GST library panning. Data are shown from individual experiments with low (0.15% Tween-20, **A**) and high (0.5% Tween-20, **B**) detergent concentration. In **B**, dark and grey boxes show results from two individual panning rounds.

Using the phage display library approach, 49 human SH3 domains displayed *in vitro *binding to hFasL(1-80) (Table 1 and Fig 2). Of note, some SH3 domains were identified only once in one of the two screenings, for instance the tyrosine kinase Itk. In contrast, for other SH3 domains, including those of CD2BP1, osteoclast stimulating factor 1 (OSTF1) and sorting nexin 33 (SNX33), several transduced clones were found. The number of hits, however, does not necessarily reflect the binding specificity of SH3 domains to the hFasL PRD, since it cannot be guaranteed that all 288 phage clones are represented in comparable amounts within the library. Of note, in the case of CD2BP1, we detected a point-mutated residue within the SH3 domain which was verified by the supplier and which might have an impact on the affinity to target motifs.

**Table 1:** GST-hFasL(1-80)-interacting SH3 domains.

**Uniprot ID**	**Entry name**	**Protein name**	**Function**	**Localization**	**Tissue**	**SH3 domain**	**Number of hits**		
							
							**total**	**0.15%**	**0.5%**
P07948	**LYN**	tyrosine protein kinase Lyn	YK, E	MA	LYM	1/1	43	20	23

O43586	PPIP1*	CD2BP1*	CA	MA	LYM	1/1	42	14	28

Q92882	**OSTF1**	osteoclast-stimulating factor 1	n.a.	CYT	LYM	1/1	42	20	22

Q8WV41	**SNX33**	sorting nexin 33	A	CYT, MA	UBI	1/1	15	4	11

P08631	**HCK**	tyrosine protein kinase Hck	YK, E	MA, CYT	LYM	1/1	14	5	9

P16333	**NCK1**	Nck1	CA	CYT, MA	LYM	2/3	11	7	4

P07947	**YES**	tyrosine protein kinase Yes	YK, E	CYT	UBI	1/1	8	2	6

P12931	**SRC**	tyrosine protein kinase Src	YK, E	CYT	UBI	1/1	8	5	3

Q9Y5X1	**SNX9**	sorting nexin 9	A	CYT, MA	LYM	1/1	6	6	0

Q7Z6B7	**SRGP1**	SLIT-ROBO Rho GAP 1	R	MA	UBI	1/1	6	3	3

A1X283	**SPD2B**	SH3 and PX domain containing protein 2B	n.a.	CYT	UBI	3/4	6	3	3

A1X283	**SPD2B**	SH3 and PX domain containing protein 2B	n.a.	CYT	UBI	4/4	5	3	2

Q06187	**BTK**	tyrosine protein kinase Btk	YK, E	CYT, MA	LYM	1/1	5	5	0

Q8TE67	**EPS8L3**	EGFR kinase substrate 8-like protein 3	A	CYT	UBI	1/1	4	3	1

Q9BRR9	**RHG09**	ARHGAP9	R	CYT, MA	LYM	1/1	4	1	3

Q9UKN7	**MYO15**	Myosin XV	CA	CYT	UBI	1/1	3	3	0

O43639	**NCK2**	Nck2	CA	CYT, MA	LYM	2/3	3	2	1

O15117	**FYB**	Fyn-binding protein	A	CYT, NUC	LYM	1/1	2	2	0

P42680	**TEC**	tyrosine protein kinase Tec	YK, E	CYT	UBI	1/1	2	2	0

P02549	**SPTA1**	spectrin alpha chain	CA	CYT	ERY	1/1	2	2	0

Q9NSI8	**SAMN1**	SAM domain-containing protein SAMSN-1	n.a.	n.a.	LYM	1/1	2	2	0

Q8TEC5	**SH3R2**	putative E3 ubiquitin protein ligase SH3RF2	E, R	n.a.	UBI	3/3	2	2	0

Q15700	**DLG2**	disks large homology 2	CA, R	MA	NL	1/1	2	1	1

O60504	**VINEX**	vinexin	CA	MA, CYT	NL	2/2	2	1	1

O75563	**SKAP-2**	Src kinase-associated phosphoprotein 2	CA	MA, CYT	LYM	1/1	2	1	1

P46108	**CRK**	proto-oncogene c-Crk/p38	A	MA, CYT	NL	2/1	1	0	1

Q9NZQ3	**SPN90**	SH3 adapter protein SPIN90	A, CA	NUC	UBI	1/1	1	0	1

Q5TCZ1	**SPD2A**	SH3 and PX domain containing protein 2A	A	CYT, MA	UBI	5/5	1	0	1

Q9UHR4	**BI2L1**	brain-specific angiogenesis inhibitor 1-associated protein 2-like protein 1	CA	CYT	UBI	3/3	1	0	1

Q99963	**SH3G2**	endophilin-A3	A	CYT, MA	UBI	1/1	1	0	1

O95049	**ZO3**	tight junction protein ZO-3	A	CYT, MA	UBI	1/1	1	0	1

P54284	**CACB3**	voltage-dependent L-type calcium channel subunit beta-3	E	MA	NL	1/1	1	0	1

Q96JB8	**MPP4**	MAGUK p55 subfamily member 4 (isoform 2)	A	CYT	UBI	1/1	1	0	1

-	**-**	N-Src	-	-	-	1/1	1	0	1

P27986	**P85A**	PI3K regulatory subunit alpha	R, A	MA	LYM	1/1	1	0	1

Q6XZF7	**DNMNP**	dynamin-binding protein	CA	MA, GOL	UBI	4/6	1	0	1

Q8N1I0	**DOCK4**	dedicator of cytokinesis protein 4	R, A	MA	NL	1/1	1	1	0

O60229	**KALRN**	kalirin	R, CA	MA	NL	1/2	1	1	0

Q6ZN28	**7A5**	SH3 domain-containing protein 7A5	TF	CYT, NUC	UBI	1/1	1	1	0

O43295	**SRGP3**	SLIT-ROBO Rho GAP 3	R	MA	NL	2/3	1	1	0

P06241	**FYN**	tyrosine protein kinase Fyn	YK, E	MA	LYM	1/1	1	1	0

Q16674	**MIA**	melanoma-derived growth regulatory protein	n.a.	SEC	NL	1/1	1	1	0

O75044	**FNBP2**	SLIT-ROBO Rho GAP 2	R	MA	NL	1/1	1	1	0

Q9NZM3	**ITSN2**	intersectin 2	A, CA	CYT	LYM	2/5	1	1	0

P14598	**NCF1**	p47phox	A	CYT	NL	1/2	1	1	0

Q08881	**ITK**	tyrosine protein kinase Itk	YK, E	MA	LYM	1/1	1	1	0

O00305	**CACB4**	voltage-dependent L-type calcium channel subunit beta-4	E	MA	NL	1/1	1	1	0

A6NJZ7	**RIM3C**	RIMS-binding protein 3C	n.a.	MA, CYT	n.a.	2/3	1	1	0

Individual interactors identified by phage display screening are listed according to their Uniprot ID, the respective entry name and the full protein name. In addition, putative protein function and subcellular localization of individual proteins are given according to the respective Uniprot entries. Primary tissue-specific expression is indicated according to the BioGPS database (Genomics Institute, Novartis Research Foundation). The number of hits is given in total and separately for the two applied detergent concentrations. SH3 domains binding to the GST control were excluded from the list.

*Point mutation detected in the provided SH3 domain of CD2BP1

Abbreviations:

Function: YK, tyrosine protein kinase; E, enzymatic acitivity; CA, cytoskeleton-associated (adapter) protein; n.a., not annotated; A, adapter protein; R, regulatory protein; TF, transcription factor

Localization: MA, membrane-associated; CYT, cytosolic; GOL, Golgi; NUC, nuclear; SEC, secreted; n.a., not annotated

Tissue distribution: LYM, lymphoid; UBI, ubiquitous; ERY, erythroid; NL, non-lymphoid; n.a., not annotated

Several of the detected interactions have been previously described. These include FasL binding to SH3 domains of non-receptor tyrosine kinases such as Fyn, Hck, Lyn, and Src. Notably, the Fyn SH3 domain has not only been the first interaction module associated with FasL [12], but Fyn and Lyn have recently been implicated in tyrosine phosphorylation of FasL associated with sorting to multivesicular bodies [18]. Furthermore, SH3 domains of the adapter proteins Nck1 and Nck2 were found to bind hFasL in our assay. In both cases, the interaction was mediated by the second SH3 domain. This is partially in line with our previous observation that Nck-binding to FasL is mediated by the second and, although less efficiently, the third SH3 domain [13,20]. The missing reactivity of the third SH3 domain in the phage display screen may be due to a lower representation of respective phages in the library. Also, SH3 domains of the NADPH oxidase subunit p47phox and the regulatory subunit of the phosphoinositide 3-kinase (PI3K) were found to bind to the FasL PRD as reported before [13]. Interactions of FasL with members of the PCH protein family have been described in different studies based on pulldown experiments, yeast two hybrid screens, and co-immunoprecipitation and co-localisation of overexpressed proteins [14,16,17]. Although we meanwhile know that several members of different PCH protein subfamilies interact with FasL, only the SH3 domains of CD2BP1 (Proline-serine-threonine phosphatase-interacting protein 1 (PSTPIP1) in mice) and SLIT-ROBO Rho GAP2 bound hFasL(1-80) in the present study. 

Phage display library screening is a convenient screening method for the elucidation of possible *in vitro *protein-protein interactions and avoids technical obstacles like for instance a correct subcellular localization of bait and prey constructs in yeast two-hybrid screening. This method, however, also has its limitations due to the distribution of individual phage clones within the library. Moreover, the use of a bacterial host may influence the folding of the respective prey protein. Also, all phage-encoded SH3-domains are fusion proteins and thus their binding specificity may be altered. We assume that these limitations hinder the identification of all FasL-SH3 domain interactions that have been previously described, for example with other PCH proteins and SNX18 (see below).

**Figure 3:**
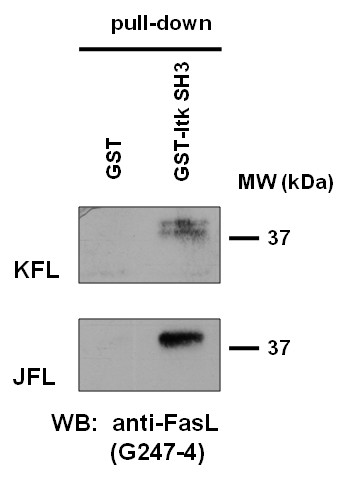
**FasL precipitates with the SH3 domain of Itk**. Pull-down experiment with a GST-Itk SH3 domain fusion protein from whole cell lysates of stably FasL transfected KFL9 and JFL39.1 cells. Lysates were subjected to pull-down experiments with the indicated recombinant proteins. Precipitates were analyzed by SDS-PAGE and Western blot for the presence of FasL using the mAb clone G247-4.

Importantly, we identified several previously not described putative interaction partners of the FasL N-terminal region. In addition to the non-receptor tyrosine kinases mentioned before, the phage display library screen revealed that the Src-like kinase Yes as well the Tec family kinases Tec and Itk might bind to hFasL. Although Yes is expressed in a number of cells types, including T cells [21], its actual involvement in T cell signal transduction and especially FasL phosphorylation still remains elusive. Tec kinases constitute a well-characterized family of non-receptor tyrosine kinases that have been implicated in a variety of signaling processes, predominantly controlled by Src kinases. In particular, the involvement of Itk in T cell development and activation has been highlighted over the past few years [22]. The interaction of Tec kinases with FasL might therefore be interesting in the context of primary T cell activation and is already subject of further investigation. In a first series of pull-down experiments, we used a GST fusion protein containing the isolated Itk SH3 domain to verify FasL binding (Fig 3). As shown before for other interactions [13], FasL was precipitated with Itk SH3 domains from lysates of stably FasL expressing KFL9 cells as a doublet and from JFL39.1 cells as a single band. Whether full length Tec family kinases also interact with FasL, and whether there is a differential binding to full length versus proteolytically processed FasL remains to be assessed.

**Figure 4:**
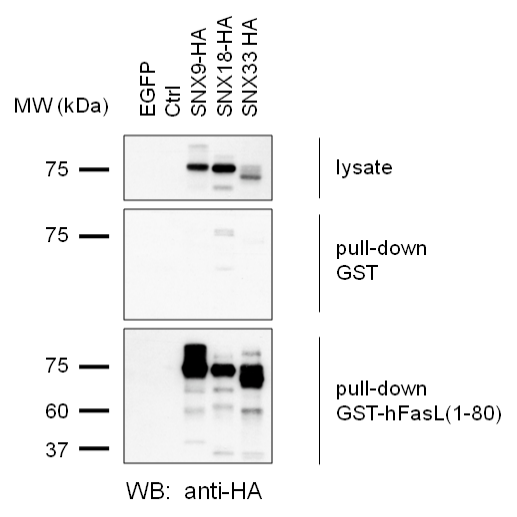
**The FasL N-terminus precipitates sorting nexin**. Pull-down experiment with GST fusion proteins containing the intracellular part of FasL from transiently transfected 293 T cells. Overexpression of sorting nexins in 293 T cells was tested 18 hours post transfection in whole cell lysates (upper panel). The corresponding lysates were subjected to precipitation with GST alone as a control or with a GST fusion protein containing the intracellular region of human FasL (hFasL(1-80)). Precipitated proteins were analyzed by SDS-PAGE and Western blotting using an anti-HA antibody. (EGFP, EGFP-transfected control cells; ctrl, vector-transfected controls).

Several previously identified FasL interactors regulate the subcellular localization of the death factor, and have been implicated in its association with the lysosomal compartment or the transport of FasL-loaded secretory vesicles to the immunological synapse [6,23]. In our phage display screen, we identified the sorting nexins SNX9 and SNX33, as well as endophilin A3 and intersectin-2 as potential binding partners for FasL. All four proteins are involved in membrane or vesicle trafficking and might therefore act as co-regulators of FasL transport in T and NK cells. The closely related SNX9 and SNX33 are members of the large sorting nexin protein family which share a phosphoinositide-binding phox-homology (PX) domain and are associated with various aspects of endocytic and endosomal sorting [24]. Together with SNX18, which was identified to interact with FasL in neuronal cells [17], SNX9 and SNX33 share a similar structural organization and are thus grouped into the SNX9 subfamily of sorting nexins [25]. The three proteins are widely expressed, even though expression levels may vary between individual tissues and cell types. In the context of FasL, the sorting nexins could play a role during internalization and processing of FasL N-terminal fragments following shedding and/or intramembrane proteolysis. In the case of the sorting nexins, we verified the interaction with the cytosolic region of FasL in pull-down experiments performed from lysates of individually transfected 293 T cells. In this case, all three HA-tagged sorting nexins (9, 18 and 33) were readily detected by an anti-HA antibody in whole cell lysates and in precipitates with GST-hFasL(1-80) fusion proteins but not in GST control precipitates (Fig 4). As for Tec kinases and PCH proteins, future experiments will address the potential differential binding of individual interactors to full length versus proteolytically processed FasL.

Endophilins are cytosolic proteins that also contain N-terminal Bin-Amphiphysin-Rvs (BAR domains) and C-terminal SH3 domains and are implicated in membrane tubulation during endocytosis [26]. Endophilin A3 enhances amyloid precursor protein (APP) α-secretase cleavage, possibly by inhibition of APP endocytosis [27]. Its role in endocytosis and APP shedding as well as its probable association with ADAM proteases could make endophilin A3 an interesting interaction partner of FasL in the context of "re-internalization". Intersectin-2 is a cytosolic adapter protein that, among other motifs, harbors five SH3 domains and a pleckstrin homology (PH) domain for membrane association. It was also implicated in clathrin-mediated endocytosis and apparently links Wiskott-Aldrich syndrome protein (WASP)-initiated actin polymerization to TCR-induced endocytic processes [28,29].

The PCH proteins SLIT-ROBO Rho GAP1, SLIT-ROBO Rho GAP2 and SLIT-ROBO Rho GAP3 are coordinators of cytoskeleton-associated membrane or organelle transport processes. Both Rho and Cdc42 GTPase-activating proteins (GAPs) were associated with migratory signal transduction in neuronal cells [30]. Similarly, the Rho GTPase-activating protein 9 (ARHGAP9), which exhibits substantial GAP activity for Rac1 and Cdc42, was discussed to regulate adhesion of hematopoietic cells to extracellular matrix proteins [31]. More recently, it was shown that ArhGAP9 binds to ERK and p38 kinases and keeps these proteins in an inactive state [32]. In view of recent data from our group showing that reverse FasL signaling leads to an inhibition of T cells associated with decreased MAPK activity [8], the putative interaction of FasL with ArhGAP9 might be relevant for this aspect of FasL biology.

The myosin XV SH3 domain also bound FasL in this phage display screen. Since myosins are actin-based motor proteins, an association with the FasL N-terminus could again suggest an impact on the transport of FasL or FasL loaded vesicles. However, as shown for T cells from myosin XV deficient mice, myosin XV seems to be dispensable for cytoxicity of murine cytotoxic T-lymphocytes (CTLs) [33]. Since it is currently still a matter of debate whether FasL is actually stored in granzyme-/perforin-loaded secretory lysosomes or in a separate cytotoxic compartment [34,35], it will be interesting to determine whether myosin XV plays a role in the Nck-dependent transport of FasL and its storage granules to the cell surface.

SPIN90 (SH3 protein interacting with Nck, 90 kDa) is a cytoskeletal adapter protein with a single SH3 domain that binds to the first and third SH3 domain of Nck via its own proline motif. It contains a putative nuclear localization signal (NLS) but is functionally associated with cytosolic (re-)organization of the actin cytoskeleton, leading, for instance, to lamellopodia formation in COS-7 cells [36]. It also associates with dynamin, WASP, and the Arp2/3 (actin-related proteins 2/3) complex and plays a key role for myofibril and sarcomere assembly. The SH3 and PX domain containing adapter protein 2B (SH3PXD2B) is a so far very poorly defined gene product. It interacts with hFasL *in vitro *via its third and its fourth SH3 domain. Only recently, this protein has been implicated in podosome formation of Src-transformed fibroblasts [37].

We also identified the SH3 domains of Fyn-binding protein (FYB)/adhesion and degranulation promoting adapter protein (ADAP) and of Src kinase-associated phosphoprotein 2 (SKAP2)/Src kinase-associated phosphoprotein 55-related (SKAP55R) as putative FasL-interacting domains. FYB/ADAP is an adaptor protein involved in TCR signaling and 'inside-out' activation of T cell integrins and is expressed in various hematopoietic cells except B cells [38-40]. Upon TCR engagement, FYB/ADAP is tyrosine-phosphorylated by Fyn and is recruited via SH2 domain binding to SLP-76 (SH2 domain containing leukocyte protein of 76 kDa) in a signaling cluster with phosphorylated linker for T cell activation (LAT) and Gads. Via an internal PRD, ADAP associates with SKAP1/SKAP55 or SKAP2/SKAP55R. This complex allows for membrane-recruitment of Rap1 which then triggers integrin activation crucial for lymphocyte extravasation as well as formation of immunological synapses. In addition, FYB/ADAP also links the TCR signaling complex to the actin cytoskeleton [38-40]. Given that hFasL(1-80) potentially interacts with FYB/ADAP as well as the SKAP2/SKAP55R SH3 domains, it will be of particular interest to test whether this interaction can also be observed in CTLs and what functional implications such an interaction might have for FasL recruitment and expression.

## Conclusion

**Figure 5:**
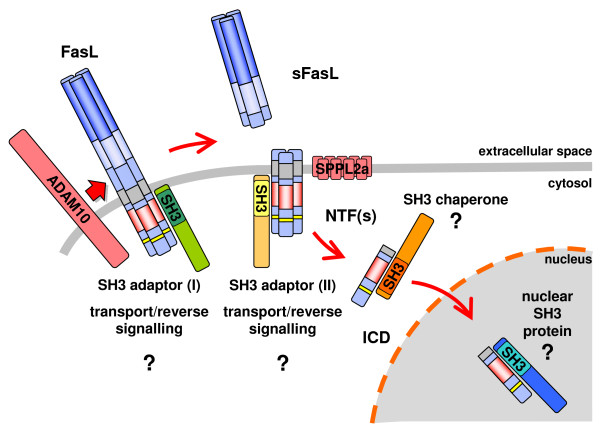
**Possible functions of FasL-interacting SH3 domain proteins**. FasL transport, storage, surface expression and reverse signaling depend on the proline-rich N-terminal region. Moreover, FasL is processed by ADAM10 generating an N-terminal fragment (NTF) and subsequently RIPped by SPPL2a generating a free intracellular domain (ICD). This ICD might translocate to the nucleus to regulate gene expression. Although several known SH3 domain proteins have been implicated in FasL storage, transport and surface expression, it is largely unknown how exactly the intracellular translocation but also the retrograde signal transduction or FasL degradation are mediated.

In conclusion, the performed phage display library screening clearly confirmed the previous notion that the N-terminal PRD of FasL is rather promiscuous in terms of SH3 binding. Meanwhile, more than 50 putative interactors have been named. Importantly, several protein families clearly stick out of the gross of SH3 domain containing proteins. These include classical cytosolic adapter proteins, non-receptor protein tyrosine kinases, PCH proteins, and sorting nexins. On the other hand, given the unusual length of the PRD, it may not be surprising to find so many potential interactors for this region. In order to understand the role of individual proteins in the context of FasL biology, we already biochemically verified the interaction of FasL with several of the novel interactors, including the Tec kinase Itk and the sorting nexins 9, 18 and 33. It is important to highlight that most if not all studies performed so far on putative FasL interactors revealed clear morphological phenotypes. We are thus confident that the present study provides a new basis for the further analysis of protein-protein interactions around FasL. Besides defining the role of individual interactions, one of the major tasks will be to determine which interaction "dominates" in a given physiological context or cell type. Here, we expect new insights into processes of endo- and exocytic vesicular transport and the modulation of signal transduction through FasL. Potential functions of newly identified SH3 domain proteins interacting with FasL are depicted in Fig 5. Given the stepwise proteolytic processing of FasL and its translocation to the nucleus, it has to be determined whether certain FasL interactors selectively regulate the "late functions" of FasL.

## Authors' contributions

MV and ML are equally contributing first authors. MV performed and evaluated all experiments. ML and OJ designed and supervised the study. OJ, ML and MV wrote the manuscript. All authors read and approved the final manuscript.
